# Anti-TLR2 antibody triggers oxidative phosphorylation in microglia and increases phagocytosis of β-amyloid

**DOI:** 10.1186/s12974-018-1281-7

**Published:** 2018-08-31

**Authors:** Ana Rubio-Araiz, Orla M. Finucane, Samuel Keogh, Marina A. Lynch

**Affiliations:** 1 Trinity College Institute for Neuroscience, Trinity College, Dublin 2, Ireland; 2 Current Address: University College Cork, Cork, Ireland

**Keywords:** Toll-like receptor 2 (TLR2), Amyloid-β (Aβ), Microglia, Neuroinflammation, Glycolysis, Oxidative metabolism, Phagocytosis

## Abstract

**Background:**

Microglia are multifunctional cells that are primarily neuroprotective and a deficit in their functional integrity is likely to be a contributory factor in the deteriorating neuronal function that occurs with age and neurodegeneration. One aspect of microglial dysfunction is reduced phagocytosis, and this is believed to contribute to the accumulation of amyloid-β (Aβ) in Alzheimer’s disease (AD). Therefore, improving phagocytosis should be beneficial in limiting the amyloidosis that characterises AD.

**Methods:**

Here, we investigated whether an antibody that targets toll-like receptor (TLR)2 might attenuate the inflammatory and metabolic changes induced by lipopolysaccharide (LPS) and amyloid-β. The impact on phagocytosis was assessed by immunohistochemistry. We evaluated the metabolic changes with the SeaHorse Extracellular Flux Analyser and studied the expression of key enzymes driving glycolysis by western blotting. For all experiments, statistical significance was determined by unpaired Student’s *t* test and two-way analysis of variance (ANOVA).

**Results:**

We have reported that, when exposed to an inflammatory stimulus, microglia switch their metabolism towards the metabolically- inefficient glycolysis; this potentially impacts on metabolically demanding functions like phagocytosis. Anti-TLR2 antibody increased phagocytosis of Aβ in LPS + Aβ-stimulated microglia and this was linked with the ability of the antibody to attenuate the LPS + Aβ-triggered inflammasome activation. LPS + Aβ increased glycolysis in microglia and increased the expression of 6-phosphofructo-2-kinase/fructose-2,6-biphosphatase (PFKFB)3, an enzyme that plays a key role in driving glycolysis; these effects were inhibited when cells were incubated with the anti-TLR2 antibody. The data also show that antibody treatment increased oxidative metabolism.

**Conclusions:**

Thus, microglia with an inflammatory phenotype, specifically cells in which the inflammasome is activated, are glycolytic; this may compromise the metabolic efficiency of microglia and thereby provide an explanation for the reduced phagocytic function of the cells. We propose that, by restoring oxidative metabolism and reducing inflammasome activation in microglia, phagocytic function is also restored.

**Electronic supplementary material:**

The online version of this article (10.1186/s12974-018-1281-7) contains supplementary material, which is available to authorized users.

## Background

The concept that microglia conform to a binary activation pattern, resting or activated, has been roundly dismissed, as has the idea that the cells adopt one of two primary activation states. Instead, microglia respond to many different stimuli in a rather specific manner with the possibility of adopting multiple different function phenotypes.

In macrophages, it has been shown that inflammatory stimuli trigger macrophages not only to adopt an inflammatory phenotype, but also to switch their metabolism towards glycolysis, thereby rapidly producing the ATP required to carry out their immune function [1–4]. Indeed, glycolysis plays a significant role in sustaining the proinflammatory roles of macrophages because certain glycolytic enzymes, including pyruvate kinase M2 (PKM2) and glyceraldehyde 3-phosphate dehydrogenase, modulate inflammatory cytokine production [5]. In addition, hexokinase 1, which catalyses the first step in glycolysis, and PKM2, can both lead to activation of the inflammasome and interleukin (IL)-1β production [6, 7].

Recent evidence from this laboratory has revealed that microglia, like macrophages, also adopt a glycolytic phenotype when challenged with an inflammatory stimulus. Specifically, interferon-γ (IFNγ) triggered activation of primary cultured microglia, increased production of inflammatory cytokines and shifted the cells towards glycolysis [8]. It has also been shown in BV2 cells that stimulation with lipopolysaccharide (LPS) + IFNγ increased glucose consumption, hexokinase activity and lactate production which are indicative of glycolysis [9], while LPS alone increased glycolysis and decreased mitochondrial metabolism also in BV2 cells [10]. Importantly, we have demonstrated that microglia prepared from transgenic mice that overexpress human amyloid precursor protein (APP) and presenilin 1 (PS1; APP/PS1 mice) had both an inflammatory and glycolytic phenotype [8] while amyloid-β (Aβ) stimulates inflammatory cytokine production in LPS-primed microglia [11].

Aβ interacts with several proteins on glia and neurons including various integrins, immunoglobulin and scavenger receptors, the neurotransmitter receptors, *N*-methyl-D-aspartate receptor and α7-nicotinic acetylcholine receptor and the receptor for advanced glycation end products (RAGE) [12]. However, it has been known for some time that it also interacts with Toll-like receptor (TLR)2, TLR4 and CD14 on microglia [13–15] triggering signalling events, including activation of nuclear factor (NF)κβ that stimulates the production of reactive oxygen species (ROS) and inflammatory cytokines. TLR2/3 deficiency attenuated the Aβ-induced changes in microglia. Consistently, our data have indicated that Aβ-induced increase in production of inflammatory cytokines was inhibited by pre-treating microglia with an anti-TLR2 antibody [16] and, interestingly, anti-TLR2 antibody prevented the Aβ-induced decrease in long-term potentiation (LTP), correlating with the evidence that the interaction of Aβ with TLR2 is associated with impaired cognition [14]. The evidence also indicates that administration of anti-TLR2 antibody to APP/PS1 mice improves cognitive function and this is associated with a decrease in microglial activation and with a decrease in Aβ accumulation, suggesting that it may affect phagocytosis [17].

The inflammasome requires two signals to ensure its activation; the first, for example, a TLR4 agonist, is responsible for upregulating mRNA expression of IL-1β and other components of the inflammasome, and the second triggers assembly of the inflammasome [18]. It has been shown that Aβ can act as signal 2 triggering assembly of the inflammasome [19]. Without the two signals, activation will not occur and therefore inhibiting one of the signals should prevent inflammasome activation. Since Aβ interacts to induce at least some of its actions by engaging TLR2, as confirmed by the finding that anti-TLR2 antibody inhibits Aβ-induced changes [16, 17], we argued that anti-TLR2 antibody would therefore inhibit the inflammasome and downstream changes.

We aimed to determine whether anti-TLR2 antibody impacts on the LPS + Aβ-induced activation of the inflammasome and therefore on the metabolic and functional phenotype in microglia. The data indicate that LPS + Aβ-induced inflammasome activation was indeed inhibited by anti-TLR2 antibody. Aβ phagocytosis by microglia was increased in LPS-treated microglia that were incubated with anti-TLR2 antibody treated and the phagocytic cells were lysosomal-associated membrane protein 1 (LAMP1)^+^ and arginase 1 (Arg1)^+^. The evidence suggests that the phagocytic capability of the cells was enhanced because the metabolic shift to glycolysis induced by LPS + Aβ was reversed by anti-TLR2 antibody, which also boosted oxidative metabolism in the cells.

## Methods

### Cell culture

Microglia were prepared as previously described [16]. Briefly, isolated mixed glia from cortical tissue of neonatal mice were cultured in T25 cm^2^ flasks in Dulbecco’s modified Eagle’s medium (cDMEM) containing foetal bovine serum (FBS), penicillin and streptomycin (100 U/ml) supplemented with macrophage colony stimulating factor (M-CSF; 100 ng/ml; R&D Systems, UK) and granulocyte macrophage colony stimulating factor (GM-CSF; 100 ng/mL; R&D Systems, UK) for 10–12 days, after which time non-adherent microglia were seeded in 24-well plates (1 × 10^5^cells/well) and cultured for a further 2 days. Medium was replaced with fresh cDMEM ± LPS (100 ng/ml; Enzo Life Sciences, UK). After 4 h, anti-TLR2 antibody (T2.5; OPN-301; a kind gift from Opsona Therapeutics; 100 nM) was added followed by the addition of Aβ (10 μM; Invitrogen, UK) 30 min later and incubation continued for 24 h; this concentration was chosen following preliminary data indicating that this concentration inhibited the LPS + Aβ-induced increase in IL-1β but did not affect IL-6 and TNFα, or cytokine mRNA expression. The incubation conditions were chosen because our preliminary and published evidence showed that the maximum effect of LPS on release of IL-1β, IL-6 and TNFα was after a 24 h incubation period and that, although some effects of Aβ were observed after 6 h incubation [16], other changes, including an increase in IL-1β mRNA, were not evident until after 24 h [20]. With respect to the period to which cells were exposed to anti-TLR2 antibody, we found that, in our experiments designed to assess the impact of anti-TLR2 antibody on Aβ-induced changes in long-term potentiation, a short pre-incubation with the antibody was optimal [16].

To prepare Aβ, lyophilized Aβ_1–40_ and Aβ_1–42_ peptides were dissolved in HPLC grade water to provide a 6 mg/ml stock solution, diluted to 1 mg/ml using sterile phosphate-buffered saline (PBS) and allowed to aggregate (24 h, 220 rpm, 37 °C). This preparation contains oligomers as evidenced by enhanced Thioflavin T binding (data not shown). Incubation continued for a further 24 h after which time cells were harvested for analysis of mRNA, immunocytochemistry, western immunoblotting or for analysis of metabolism using Seahorse technology. In some experiments, the effect of inhibiting oxidative metabolism was assessed by including rotenone in the incubation medium (0.05 μM) for 2 h prior addition of anti-TLR2 antibody.

### Analysis of cytokines by ELISA and RT-PCR

Cytokine concentrations were assessed in supernatant samples from cultured microglia [21]. Briefly, 96-well plates (Nunc-Immuno plate with Maxisorp surface, Denmark) were coated with capture antibody (rat anti-mouse IL-1β or TNFα antibody (R&D Systems, USA) and incubated (overnight, room temperature). Duplicate samples or standards (50 μl/well) were added and plates were incubated (24 h, 4 °C) and washed before addition of detection antibody (biotinylated goat anti-mouse; 1 h, room temperature). Plates were washed, incubated with streptavidin-horseradish peroxidase conjugate (20 min, room temperature) and washed before addition of substrate solution (50 μl; 1:1 hydrogen peroxide (H_2_O_2_): tetramethylbenzidine; R&D Systems, USA). After colour development, the reaction was stopped by adding 1 M sulphuric acid (H_2_SO_4_; 25 μl) and plates were read at 450 nm (Labsystem Multiskan RC, UK). Cytokine mRNA expression was assessed in harvested microglia by RT-PCR as previously described [22].

### Western immunoblotting

Microglia were prepared for western immunoblotting as described [11]. Briefly, cells were incubated in lysis buffer (composition in millimolar: Tris-HCl 10, NaCl 50, Na_4_P_2_O_7_.H_2_O 10, NaF 50, containing 1% each of Igepal, phosphatase inhibitor cocktail I and II, and protease inhibitor; Sigma, UK), equalised for protein, added to 4× SDS sample buffer (composition: Tris-HCl 100 mM, pH 6.8, 4% SDS, 2% bromophenol blue, 20% glycerol; Sigma, UK), boiled (100 °C, 5 min) and applied to 10% SDS gels. Proteins were transferred to nitrocellulose membrane, non-specific binding was blocked and membranes were incubated overnight at 4 °C with the antibodies raised against PFKFB1 or PFKFB3 (both IgG, raised in rabbit, 1:1000 in 5% non-fat dried milk/TBS-T; Abcam, USA). Membranes were washed and incubated (room temperature, 2 h) with a secondary HRP-linked anti-rabbit antibody (1:5000 in 5% milk in TBS-T). Immunoreactive bands were detected using WesternBright ECL chemiluminescent substrate (Advansta, USA). Images were captured using the Fujifilm LAS-4000 imager, and densitometric analysis was carried out using ImageJ (http://rsb.info.nih.gov/).

### Metabolic analysis

The SeaHorse Extracellular Flux (XF24) Analyser (SeaHorse Bioscience, USA) was used to carry out bioenergetic analysis of cells. Microglia (1 × 10^5^cells/well) were seeded (100 μl/well) on SeaHorse cell culture microplates and incubated in cDMEM ± LPS (100 ng/ml; Enzo Life Sciences, UK). After 4 h, anti-TLR2 antibody (100 nM) was added followed by the addition of Aβ (10 μM; Invitrogen, UK) 30 min later. The sensor cartridge was hydrated by adding SeaHorse XF Calibrant solution (1 ml; SeaHorse BioScience, USA) to each well of the utility plate and left overnight in a CO_2_-free incubator at 37 °C. Following incubation, cells were washed twice with the assay medium for the mitochondrial stress test or the glycolytic flux test (1 ml) according to the manufacturer’s instructions, specific assay media was added to give a final volume of 475 μl/well and the plate was incubated in a CO_2_-free incubator (37 °C, 1 h). For the mitochondrial stress test, oligomycin (20 μM; Abcam, UK), carbonyl cyanide-4-(trifluoromethoxy)phenylhydrazone (20 μM; FCCP; Sigma-Aldrich, UK) and antimycin A (40 μM; Sigma-Aldrich, UK) were loaded into the appropriate ports for sequential delivery. For the glycolytic flux test, glucose (10 mM), oligomycin (20 μM) and 2-deoxy-D-glucose (2-DG; 500 mM; all Sigma-Aldrich, UK) were prepared in glycolytic flux assay media and similarly loaded into the appropriate ports. Following calibration, oxygen consumption rate (OCR) and extracellular acidification rate (ECAR) were measured every 8 min for 96 min and the appropriate compounds were injected sequentially at 24 min intervals. ECAR and OCR were automatically calculated using the SeaHorse XF24 software and 4–6 replicates were assessed for each separate sample.

### Confocal image analysis

Microglial cells, plated onto poly-D-lysine-coated cover slips, were fixed with paraformaldehyde (PFA) (4%; 30 min), washed and blocked by incubation with 10% NHS and 0.1% Triton X-100 for 1 h and incubated at 4 °C with the primary antibodies anti-Iba1 (Wako, Japan 1:1000), anti-Aβ 6E10 (Biolegend, USA, 1:500), anti-LAMP1 (Abcam, UK, 1:250) and anti-Arginase (Arg)1 (Santa Cruz, USA, 1:500) followed by the secondary antibodies Alexa Fluor® 594 donkey anti-rabbit IgG (1:1000), Alexa Fluor® 488 donkey anti-mouse IgG (1:1000) and Alexa Fluor® 633 donkey anti-goat IgG (1:1000) and mounted in ProLong®Gold with the nuclear marker 4′,6-Diamidine-2′-phenylindole dihydrochloride (DAPI; Thermo Scientific, USA). Images (10 fields per experiment, triplicate analysis; × 40 magnification) were acquired with a Zeiss AX10 Imager A1 microscope. Analysis of images was undertaken with ImageJ software; the number of Aβ^+^ microglia was assessed. Control experiments with each secondary antibody, alone or in combination, were included and images were acquired sequentially on a Leica SP8 scanning confocal microscope for each fluorophore to eliminate cross-signal contamination. Analysis revealed that anti-TLR2 antibody staining of Iba1^+^ cells was confined to the membrane (see Additional file 1: Figure S2).

### Analysis of Aβ engulfment by microglia

For analysis of the effect of LPS on phagocytosis, microglia (1 × 10^5^cells/well were plated onto coverslips coated with poly-D-lysine (5 μg/ml; Merck Millipore Ltd., UK). After 48 h, cells were preincubated with LPS (100 ng/ml; Ezo Life Sciences, UK) for 4 h then stimulated for 24 h with Aβ_1–40_ and Aβ_1–42_ peptides (5 μM; Invitrogen, UK), and pulse-centrifuged for 15 s in a benchtop microcentrifuge. This procedure removed large aggregates and microscopic analysis indicated that that the preparation comprised mainly oligomers. The ratio of Iba^+^ cells that engulfed Aβ was calculated by enumerating the total number of Iba^+^ cells and the number of Iba^+^ Aβ^+^ cells in 3D projection images generated from Z stacks using a Leica SP8 scanning confocal microscope and processed using LAS AF Lite software. A total of 10 fields per experiment in triplicate were analysed.

### Caspase 1 activity assay

To analyse the activity of caspase 1 in microglia, we used a commercially available Caspase-1/ICE Colorimetric Assay (R&D Systems) and we assessed activity in cultured microglia, which were collected by centrifugation (250×*g*, 10 min) in a conical tube. The supernatant was gently removed and discarded while the cell pellet was lysed by the addition of cold lysis buffer (25 μl/1 × 10^6^ cells). The cell lysate was incubated (4 °C, 10 min) and centrifuged (10,000×*g*, 1 min). The enzymatic reaction for caspase activity was carried out in a 96-well flat-bottomed microplate with cell lysate (50 μl), 2X Reaction Buffer+ 1% DTT (50 μl) and caspase-1 colorimetric substrate (5 μl, YVAD-pNA). The plate was incubated at 37 °C for 2 h and read on a microplate reader at a wavelength of 405 nm. The results are expressed as fold increase in caspase 1 activity in treated cells relative to control cells.

### Statistical analysis

Data are reported as the mean ± SEM and the number of experiments is indicated in each case. Statistical analysis was carried out using a one-way analysis of variance (ANOVA), with Newman-Keuls multiple comparison test. When comparisons were being made between two conditions, an unpaired Student’s *t* test was performed. The significance level was set at *p* < 0.05.

## Results

**Fig. 1:**
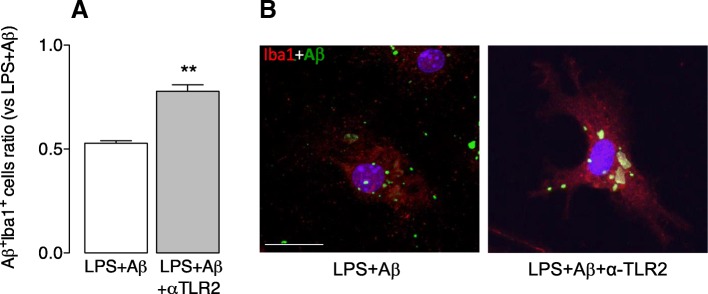
Anti-TLR2 antibody increases phagocytosis of Aβ**.** Microglia from neonatal mice were plated on poly-D-lysine-coated coverslips, incubated with LPS (100 ng/ml) for 4 h after which time anti-TLR2 antibody (2.5 mM/ml) was added. Aβ (10 μM) was added 4.5 h later and incubation continued for 24 h. Cells were harvested, fixed in 4% PFA and stained for Iba1 and Aβ as described in the “Methods” section. **a** Mean data revealed that incubation of LPS + Aβ-stimulated cells with anti-TLR2 antibody significantly increased phagocytosis of Aβ (**p < 0.01 LPS + Aβ+anti-TLR2 vs LPS + Aβ; Student’s *t* test for independent means; *n* = 3). Aβ uptake is expressed as a ratio between the number of Aβ^+^ Iba^+^ cells as a total number of Iba^+^ cells. A total of 10 fields per experiment in triplicate were analysed. **b** The panel shows confocal fluorescence images at × 63 magnification. The presence of Aβ (green) is evident in Iba1^+^ (red) cells that were incubated in LPS + Aβ with anti-TLR2 antibody. (Scale bar = 20 μm)

We assessed phagocytosis of Aβ in microglia treated with LPS in the presence/absence of anti-TLR2 antibody and show that cells incubated with LPS + Aβ and anti-TLR2 antibody exhibited an increase in Aβ engulfment compared with cells incubated with LPS + Aβ (**p < 0.01; Student’s *t* test for independent means; Fig. 1a, b).

**Fig. 2:**
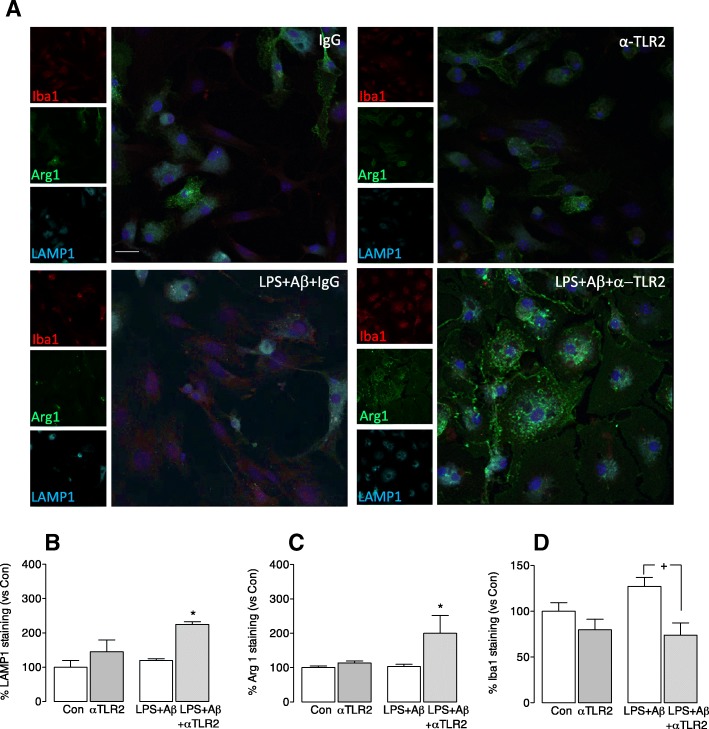
Anti-TLR2 antibody increases the expression of Arg1 and LAMP1 and decreases Iba1 expression in microglia. Microglia were prepared and treated as described in the legend for Fig. 1 and stained for LAMP1, Arg1 and Iba1 as described in the “Methods” section. **a** The panel shows confocal fluorescence images at × 40 magnification and shows that Iba1 staining was increased in microglia incubated with LPS + Aβ. LAMP1 staining and Arg1 staining were increased in microglia that were incubated in LPS + Aβ+anti-TLR2 antibody. (Scale bar = 50 μm). **b**–**d** Analysis of the mean data indicate that incubation of LPS + Aβ-stimulated cells with anti-TLR2 antibody significantly increased LAMP1 staining (**b**) and Arg1 staining **c**, whereas the significant LPS + Aβ-induced increase in Iba1 (**d**) was attenuated when cells were also incubated with the antibody ((F3,11) = 4.53; *p* = 0.033 one-way ANOVA *p < 0.05; control vs LPS + Aβ; ^+^*p* < 0.05: Newman-Keuls multiple comparison test; LPS + Aβ vs LPS + Aβ+anti-TLR2 antibody). Data are expressed as the percentage of LAMP (**a**), Arg1 (**b**) or Iba1 (**c**) staining in each treatment group vs the control. A total of 10 fields per experiment in triplicate were analysed

The lysosomal protein LAMP1 was increased in cells incubated with LPS + Aβ+anti-TLR2 antibody compared with those incubated with LPS + Aβ (*p < 0.05; Fig. 2b). A similar marked increase in Arg1 was observed in cells incubated with LPS + Aβ+anti-TLR2 antibody compared with cells incubated with LPS + Aβ (*p < 0.05; Fig. 2c). It can be inferred from the co-localization of Arg1 and LAMP1 (Fig. 2a) that Arg1^+^ cells are effective phagocytes. The increased expression of LAMP1 and Arg1 on cells was associated with a decrease in expression of the microglial marker Iba1 which is upregulated in inflammatory microglia. The data show specifically that Iba1 fluorescence was significantly decreased in cells that were incubated with LPS + Aβ+anti-TLR2 antibody compared with those incubated with LPS + Aβ (*p < 0.05; Fig. 2d). The cells incubated with LPS + Aβ+anti-TLR2 antibody also reflects a morphological change that is consistent with an increase in their phagocytic capacity; they are larger and more amoeboid.

**Fig. 3:**
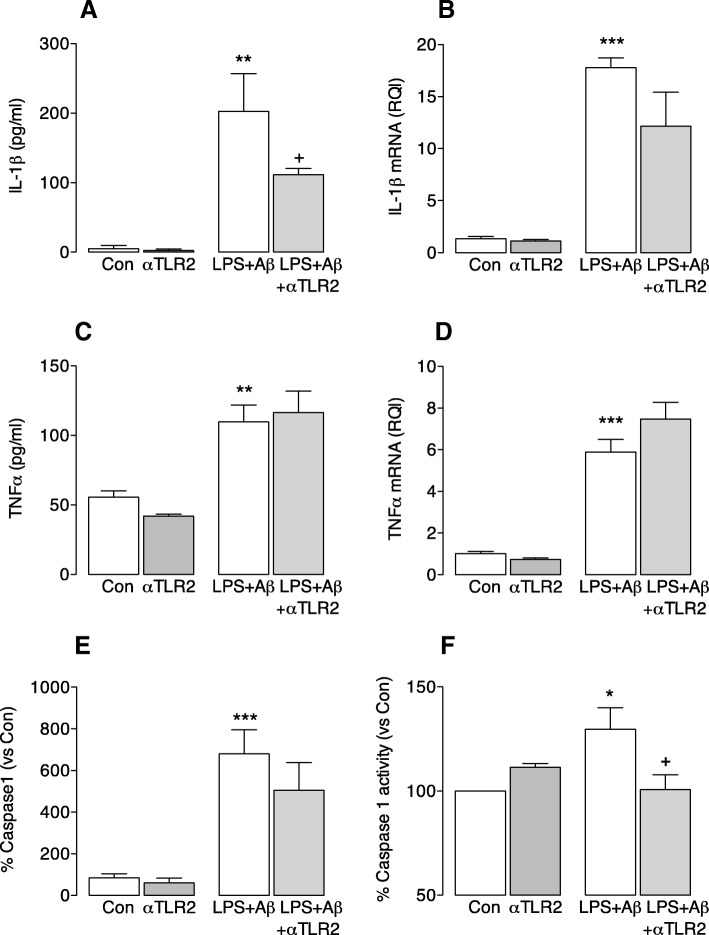
Anti-TLR2 antibody decreases IL-1β secretion from microglia. Microglia were prepared as described in the “Methods” section. Cells were harvested for analysis of IL-1β and TNFα mRNA by RT-PCR, or for staining of caspase 1, and supernatant samples were collected and assessed for cytokine concentration. Cell lysate was prepared for analysis of caspase 1 activity using a commercially available kit. **a**–**d** LPS + Aβ significantly increased supernatant concentrations of IL-1β (**a**) and TNFα (**c**) and also IL-1β mRNA (**b**) and TNFα mRNA ((**a**, (F3,14) = 10.87; *p* = 0.0010; **c**, (F3,10) = 3.386; *p* = 0.0745; **b**, (F3,16) = 11.98; *p* = 0.0004; **d**, (F3,18) = 17.10; *p* < 0.0001 one-way ANOVA **p < 0.01; ***p < 0.001; Newman-Keuls multiple comparison test). Anti-TLR2 antibody significantly attenuated the LPS + Aβ-induced increase in IL-1β but did not affect the changes in IL-1β mRNA, TNFα or TNFα mRNA. **e** LPS + Aβ significantly increased caspase 1 expression and activity but was not attenuated after anti-TLR2 treatment ((F3,32) = 10.11; *p* < 0.0001 one-way ANOVA ***p < 0.001). **f** Caspase 1 activity was increased by LPS + Aβ (*p < 0.05), and anti-TLR2 antibody attenuates this LPS + Aβ-induced effect (^+^*p* < 0.05; LPS + Aβ vs LPS + Aβ+anti-TLR2 antibody; ((F3,10) = 4.874; *p* = 0.0326 one-way ANOVA; Newman-Keuls multiple comparison test). For **e** and **f**, data are expressed as the percentage of the caspase 1 expression and activity in each treatment group vs the control; three independent experiments were performed

Anti-TLR2 antibody significantly attenuated the LPS + Aβ-induced increase in IL-1β (**p < 0.001; ANOVA; LPS + Aβ vs control; ^+^*p* < 0.05; LPS + Aβ vs LPS + Aβ+anti-TLR2 antibody; Fig. 3a). In contrast, while LPS + Aβ increased IL-1β mRNA, TNFα mRNA and TNFα secretion (**p < 0.01; ***p < 0.001; ANOVA; Fig. 3b–d), anti-TLR2 antibody exerted no modulatory effect and therefore there was a significant difference between the samples obtained from cells that were incubated with anti-TLR2 antibody alone compared with the LPS + Aβ-treated cells that were incubated with anti-TLR2 antibody (*p* < 0.01; ANOVA; anti-TLR2 antibody vs LPS + Aβ+anti-TLR2; Fig. 3b–d). The specific action of anti-TLR2 antibody on IL-1β secretion suggests that it may inhibit assembly of the inflammasome and to explore this further we assessed caspase 1 activity. LPS + Aβ significantly increased caspase 1 immunoreactivity (***p < 0.001; ANOVA; Fig. 3e), but this was not modulated when cells were incubated in the presence of anti-TLR2 antibody. However, caspase 1 activity, assessed using a fluorometric assay, was increased by LPS + Aβ (*p < 0.05) and anti-TLR2 antibody attenuated this LPS + Aβ-induced effect (^+^*p* < 0.05; LPS + Aβ vs LPS + Aβ+anti-TLR2 antibody; Fig. 3f). These data suggest that the phagocytic potential of microglia is diminished when the inflammasome is activated.

**Fig. 4:**
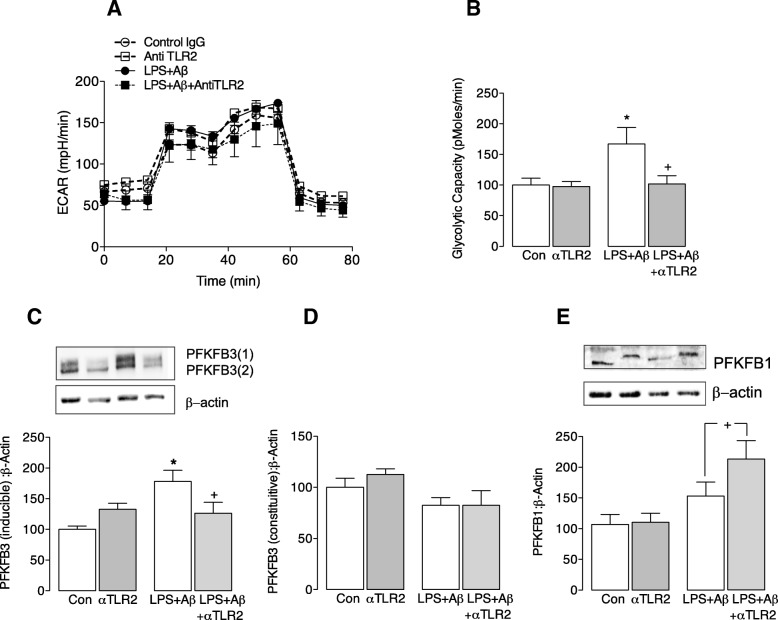
Anti-TLR2 antibody attenuates the LPS + Aβ-induced glycolytic capacity of microglia. Microglia (1 × 10^5^cells/well) were seeded (100 μl/well) on SeaHorse cell culture microplates and incubated in cDMEM ± LPS (100 ng/ml; Enzo Life Sciences, UK). After 4 h, anti-TLR2 antibody (100 nM) was added followed by the addition of Aβ (10 μM; Invitrogen, UK) 30 min later, as described in detail in the “Methods” section. **a**. The bioenergetic profile of microglia consisting of three baseline measures of ECAR followed by sequential measures following exposure to glucose (10 mM), oligomycin (20 μM) and 2-deoxy-D-glucose (2-DG; 500 mM) is shown. **b** LPS + Aβ significantly increased mean glycolytic capacity (*p < 0.05), and this was significantly attenuated when cells were also incubated with anti-TLR2 antibody (^+^*p* < 0.05; LPS + Aβ vs LPS + Aβ+anti-TLR2 antibody; (F3,13) = 4.55; *p* = 0.026; one-way ANOVA; Newman-Keuls multiple comparison test). **c**, **d**. LPS + Aβ significantly increased the inducible (**c**) but not the constitutive (**d**) form of PFKFB3 (*p < 0.05), and anti-TLR2 antibody significantly attenuated the LPS + Aβ-induced change (^+^*p* < 0.05; LPS + Aβ vs LPS + Aβ+anti-TLR2 antibody; (F3,18) = 6.067; *p* = 0.0059 one-way ANOVA, Newman-Keuls multiple comparison test). **e** LPS + Aβ did not affect PFKFB1 but incubated with LPS + Aβ+anti-TLR2 antibody significantly increased PFKFB1 (^+^*p* < 0.05; LPS + Aβ vs LPS + Aβ+anti-TLR2 antibody; (F3,18) = 4.594; *p* = 0.0167 one-way ANOVA, Newman-Keuls multiple comparison test). Representative immunoblots are shown. For **c**–**e**, data are expressed as the percentage of the protein expression in each treatment group vs the control; three independent experiments were performed

Recent data from this laboratory has indicated that inflammatory microglia switch their metabolism to glycolysis, and we considered that such a switch, which effectively reduces ATP production, might negatively impact on phagocytic function. The data indicate that LPS + Aβ increased glycolytic capacity (*p < 0.05; ANOVA; Fig. 4b), and this increase was attenuated by anti-TLR2 antibody (^+^*p* < 0.05; LPS + Aβ vs LPS + Aβ+anti-TLR2 antibody). In parallel with the increase in glycolytic capacity, LPS + Aβ significantly increased the inducible, but not the constitutive, form of PFKFB3 (*p < 0.05; ANOVA; Fig. 4c, d) and this was also attenuated by anti-TLR2 antibody (^+^*p* < 0.05; LPS + Aβ vs LPS + Aβ+anti-TLR2 antibody). We investigated the effect of Aβ and LPS alone on metabolism in microglia. Both slightly increased glycolysis and glycolytic capacity (see Additional file 2: Figure S1) but neither alone triggered a significant change, while the combination of LPS + Aβ significantly increased glycolysis and glycolytic capacity.

**Fig. 5:**
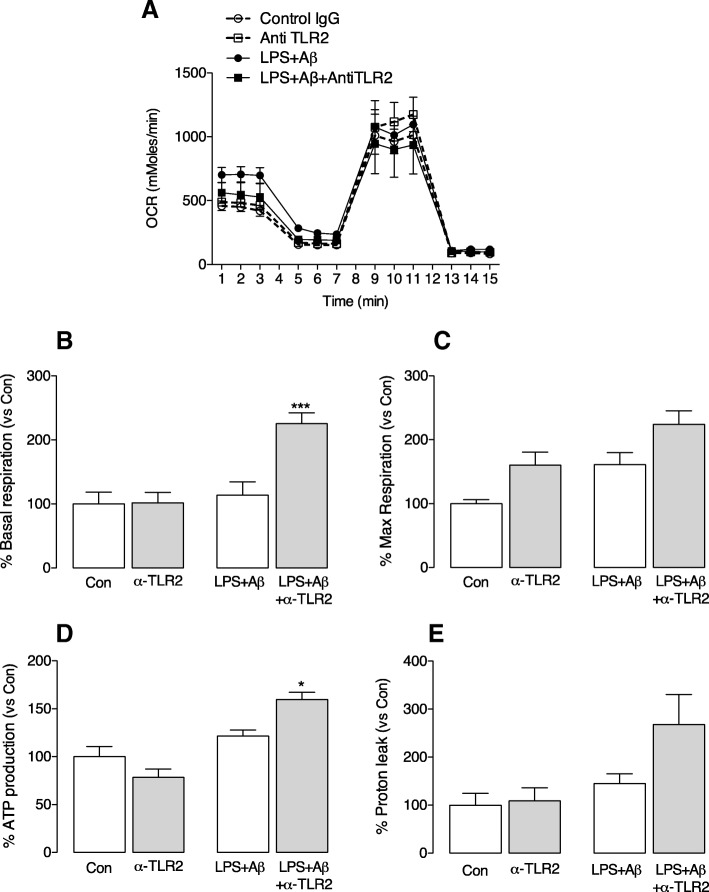
Anti-TLR2 treatment triggers oxidative phosphorylation. Primary microglia were prepared for metabolic analysis as described in the legend to Fig. 4 and in the “Methods” section. **a** The OCR bioenergetics profile consisting of three baseline measures of OCR followed by sequential measures following exposure to oligomycin (20 μM), carbonyl cyanide-4-(trifluoromethoxy) phenylhydrazone (20 μM; FCCP) and antimycin A (40 μM) is shown. **b**–**e** LPS + Aβ exerted no significant effect on mean basal respiration **b**, mean maximal respiration (**c**) or mean ATP production (**d**) or mean proton leak (**e**) but, except for maximal respiration and proton leak, each measure was significantly increased when cells were incubated in LPS + Aβ+anti-TLR2 antibody (***p < 0.001, *p < 0.05, ^+^*p* < 0.05; LPS + Aβ vs LPS + Aβ+anti-TLR2 antibody; (F3,16) = 9.666; *p* = 0.0010; one-way ANOVA, Newman-Keuls multiple comparison test); three independent experiments were performed

Anti-TLR2 antibody also increased PFKFB1 (^+^*p* < 0.05; LPS + Aβ vs LPS + Aβ+anti-TLR2 antibody; Fig. 4e) and caused a shift in molecular weight. This might reflect phosphorylation on Ser32, which has been shown to modulate its activity resulting in a decrease in glycolytic flux [23]. Significantly, analysis of OCR indicated that anti-TLR2 antibody also increased basal respiration and ATP production (***p < 0.001, ^*^p < 0.05; LPS + Aβ vs LPS + Aβ+anti-TLR2 antibody; ANOVA; Fig. 5) although neither the antibody alone nor LPS + Aβ in the absence of the antibody exerted an effect on these measures.

**Fig. 6:**
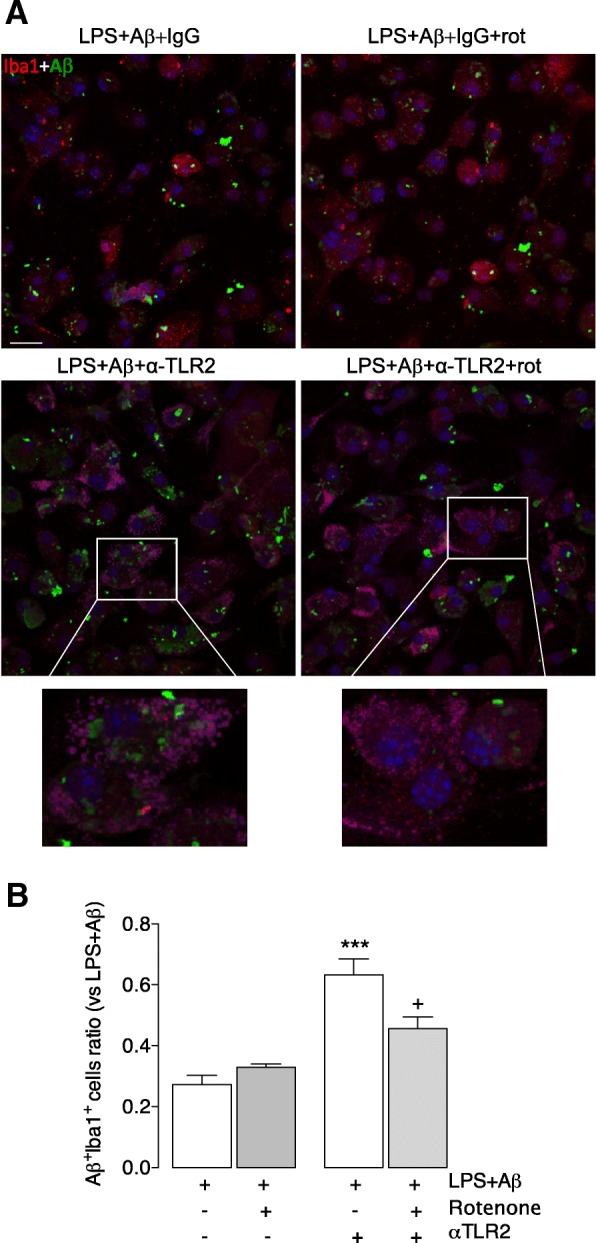
Rotenone, an inhibitor of oxidative metabolism, overcomes the stimulatory effect of anti-TLR2 antibody on phagocytosis of Aβ. Microglia were prepared as described in the legend for Fig. 1 and treated with LPS + Aβ in the presence or absence of anti-TLR2 antibody and the presence or absence of rotenone (rot). Cells were stained for Aβ and Iba1 as described in the “Methods” section. **a** The panel shows confocal fluorescence images at × 40 magnification. Sample images are presented that reveal the presence of Aβ (green) in Iba1^+^ (red) in cells that were incubated in LPS + Aβ with anti-TLR2 (left hand images) in the presence or absence of rotenone (rot; right hand images). The inserts highlights Aβ uptake by an Iba1^+^ cell. (Scale bar = 50 μm). **b** Incubation of microglia in the presence of LPS + Aβ+anti-TLR2 antibody significantly increased phagocytosis of Aβ ((F3,21) = 19.27; *p* = 0.0378 **p < 0.01; one-way ANOVA; Newman-Keuls multiple comparison test), and this effect is significantly attenuated when rotenone was added to the incubation (^++^*p* < 0.01; LPS + Aβ+anti-TLR2 antibody vs LPS + Aβ+anti-TLR2 antibody+rotenone. Aβ uptake is expressed as a ratio between the number of Aβ^+^ Iba^+^ cells as a total number of Iba^+^ cells. A total of 10 fields per experiment in triplicate were analysed

To assess whether the metabolic switch caused by anti-TLR2 antibody is involved in the augmentation of phagocytic capacity of microglia, cells stimulated with LPS + Aβ ± anti-TLR2 antibody were treated with rotenone, which inhibits complex I in the electron transport chain. We evaluated phagocytosis of Aβ in microglia in the presence/absence of rotenone and show that cells incubated with LPS + Aβ+ anti-TLR2 antibody and rotenone exhibited a decrease in Aβ engulfment compared with cells incubated with LPS + Aβ+α-TLR2 antibody (***p < 0.001, ^++^*p* < 0.01; ANOVA; Fig. 6a, b).

## Discussion

The significant finding from these experiments is that phagocytosis of Aβ is compromised in glycolytic microglia in which the inflammasome is activated by LPS + Aβ and that anti-TLR2 antibody increases Aβ phagocytosis, which we propose is a consequence of its ability to switch off inflammasome activation and to reprogramme the cells towards the metabolically more efficient oxidative phosphorylation.

The finding that anti-TLR2 antibody increased LPS + Aβ-triggered phagocytosis of Aβ by microglia is consistent with our observation that treatment of APP/PS1 mice with the antibody twice monthly for 7 months from 7 to 14 months of age reduced Aβ accumulation [17]. It is also broadly in agreement with previous findings that indicated an increase in Aβ phagocytosis by primary microglia prepared from TLR2-deficient mice [15]. Anti-TLR2 antibody also attenuated the LPS + Aβ-induced increase in Iba1, which confirms that it exerts a modulatory effect on microglial activation. Treatment of APP/PS1 mice with anti-TLR2 antibody reduced CD68 immunoreactivity in the hippocampus providing further evidence of its modulatory effect on microglial activation [17].

There is strong evidence that Aβ-induced inflammatory changes are mediated by TLR2; indeed, the critical domain on TLR2 that enables Aβ binding, and signalling that drives inflammation, has been identified as the amino acids EKKA (741–744) in the cytoplasm [15]. Whether this domain also controls phagocytosis of Aβ remains to be explicitly determined. However, it has been shown that activation of TLR2 induced phagocytosis of Aβ in microglia [24] while TLR2 deletion in APP/PS1 mice delayed Aβ plaque formation [15, 25]. In addition to TLR2, Aβ binds to other receptors that have been proposed to impact on phagocytosis. Fibrillar Aβ binds to a complex that contains CD36, CD47 and α6β1-integrin, and this binding triggers engulfment of Aβ in BV2 cells [26], a role for triggering receptor expressed on myeloid cells 2 (TREM2) in phagocytosis has also been described [27] and it has been proposed that Fc receptors [28] and TLR4 [29] are capable of phagocytosing Aβ deposits. Here, we show that the lysosomal membrane protein, LAMP1 is markedly increased in LPS + Aβ-stimulated cells that were incubated with anti-TLR2 antibody suggesting an effect of the antibody on lysosomal function. However, we are unaware of any experimental data that directly link an increase in LAMP1 expression in microglia with the phagocytic capability of cells.

LPS and Aβ together provide the two signals required to activate the inflammasome; LPS triggers signalling through the TLR4-NFκB pathway to increase mRNA expression of IL-1β and the other components of the inflammasome, while Aβ induces assembly of the inflammasome. Therefore, as predicted, LPS + Aβ increased IL-1β release and caspase 1 activation. Both LPS + Aβ-induced changes were attenuated when cells were incubated in the presence of anti-TLR2 antibody, and it is suggested that this occurs because the antibody blocked one of the two signals, Aβ, required to trigger inflammasome activation. Clearly anti-TLR2 antibody will not inhibit TLR4 activation by LPS. Whereas LPS + Aβ also increased IL-1β mRNA, as well as TNFα mRNA and TNFα release, anti-TLR2 antibody did not modulate these changes. This implies that the antibody specifically inhibits the inflammasome rather than exerting a general anti-inflammatory effect. Significantly, we have reported that inhibiting the inflammasome with the small molecule inhibitor, MCC950, increased phagocytosis of Aβ by microglia [11]. A link between phagocytosis and inflammasome activation has also been described in macrophages whereby phagocytosed pathogens can trigger activation by microbial proteins or nucleic acids, or by altering K^+^ efflux [30]. In microglia, Aβ phagocytosis triggers inflammasome assembly because of the subsequent release of cathepsin B from lysosomes [19]. The current finding indicating that inhibiting the inflammasome with anti-TLR2 antibody is paralleled by increased phagocytosis of Aβ suggests that inflammatory microglia are less phagocytic. On the contrary, the microglia that engulf Aβ are Arg1^+^, which is generally thought to be an indicator of an anti-inflammatory phenotype; this is consistent with previous findings [31]. Increased arginase 1 suggests that ornithine will ultimately be increased and this is interesting because, at least in macrophages, increased ornithine, which is a characteristic of IL-4-treated cells, is associated with increased phagocytic activity [32].

There are very few reports in the literature that have assessed the impact of inflammatory stimuli on microglial phagocytosis of Aβ specifically. Those that exist present a conflicting narrative. On the one hand, LPS increased uptake of Cy3-Aβ_42_ in microglia as assessed by evaluating the mean fluorescence values for cell-associated Cy3-Aβ_42_ while the extracellular signal was decreased [33]. In contrast, LPS and inflammatory cytokines decreased phagocytosis of microspheres in BV2 cells and this effect was inhibited by anti-inflammatory cytokines [34]. Conflicting data have also been generated from in vivo experiments. A single intrahippocampal injection of LPS in Tg2576 APP mice decreased the Aβ burden 3, 7 and 14 days after injection, but not 28 days after injection [35]. However, an earlier study indicated that intraventricular infusion of LPS for 2 weeks exacerbated Aβ deposition in transgenic mice that overexpressed APP and apolipoprotein E [36].

Here we show that LPS + Aβ increased glycolysis in microglia, consolidating our recent finding in microglia [8] and other findings in BV2 cells [9, 10], and showing that inflammatory stimuli exert this action in microglia as they do in macrophages [1]. A significant finding of the current study is that anti-TLR2 antibody attenuated the LPS + Aβ-induced effect on glycolysis and also on 6-phosphofructo-2-kinase/fructose-2,6-biphosphatase (PFKFB3), a bifunctional enzyme with predominantly kinase activity that leads to an increase in phosphofructokinase activity and thereby drives glycolysis [37–39]. Interestingly, the impact of the antibody was observed on the inducible form of the enzyme and not the constitutive form. The possibility is that this reflects phosphorylation of PFKFB1. It has been shown that phosphorylation of PFKFB1 on ser32 modulates its activity, decreasing glycolytic flux [23]. This is consistent with our finding that anti-TLR2 antibody, which induces the molecular weight shift in PFKFB1, increases oxidative metabolism.

It has been shown that glycolytic enzymes impact on the inflammasome in macrophages [40]. Hexokinase 1 interacts with voltage-dependent anion channel (VDAC)1 which activates the inflammasome while PKM2 triggers inflammasome activation in a HIF-1α-dependent manner [41] and also activates eukaryotic translation initiation factor 2A kinase, which directly interacts with inflammasome components regulating its assembly [42]. Therefore, it is possible that LPS + Aβ triggers glycolysis and, as a result, feeds back to activate the inflammasome. However, LPS + Aβ-induced glycolysis is attenuated in macrophages from NLRP3^−/−^ mice (Finucane et al. pers. comm), indicating that activation of the inflammasome drives signals that induce glycolysis, and this is consistent with the observation that IL-1β also stimulates glycolysis. Similarly, the primary modulatory effect of anti-TLR2 antibody on LPS + Aβ-induced changes may be on the metabolic profile of the cells and, in this context, it is interesting that oxidative metabolism was increased in LPS + Aβ-treated cells that were incubated with the antibody.

Glycolysis is metabolically inefficient, producing only 2 molecules of ATP per glucose, compared with oxidative metabolism which yields ~ 30 molecules of ATP. Therefore, a persistent bias towards glycolysis may have a detrimental effect on metabolically expensive processes like phagocytosis. We demonstrate that phagocytosis of Aβ is enhanced in microglia when oxidative metabolism is sustained, and when the LPS + Aβ-induced shift towards glycolysis is prevented, by anti-TLR2 antibody. This finding is consolidated by the observation that rotenone, which blocks oxidative metabolism, neutralises the beneficial effect of anti-TLR2 antibody on phagocytosis in LPS + Aβ-treated microglia.

Disrupted metabolism, including decreased glucose utilisation and mitochondrial dysfunction, are recognised features of AD [43]. Since neurons utilise most of the energy required by the brain [44] studies have tended to focus on metabolic changes in these cells. However, as microglia are arguably the primary neuroprotective cell in the brain, disruption in their metabolic profile is likely to contribute to disease. A significant finding in this study is that microglia shift towards a glycolytic phenotype in response to an inflammatory stimulus. A persistent reliance on glycolysis in microglia, because it is metabolically inefficient, will inevitably lead to reduced function, including phagocytosis, and may ultimately contribute to changes such as amyloid accumulation in AD. In this context, it is interesting that microglia from APP/PS1 mice exhibit a glycolytic phenotype [8].

One of the aims of this study was to investigate the mechanism by which the accumulation of Aβ in APP/PS1 mice was attenuated by anti-TLR2 antibody [17]. The data presented suggest that the antibody increases Aβ phagocytosis by microglia and that this is a consequence of its action on the metabolic profile of microglia. It is significant that the phagocytic microglia are Arg1^+^ suggesting that they have an antiinflammatory phenotype, and this is also suggested by the fact that the antibody-driven increase in phagocytosis is associated with a decrease in inflammasome activation. We have shown that inhibiting the inflammasome with MCC950 increased phagocytosis of Aβ by microglia and decreased Aβ accumulation in hippocampus of APP/PS1 mice. We propose that the metabolic signature of microglia impacts on their inflammatory status and on their functional integrity, and it remains to be determined whether blocking the actions of Aβ with anti-TLR2 antibody in APP/PS1 mice will primarily affect the metabolic profile and consequently restore cell function. 

## Conclusions

The data demonstrate that targeting TLR2 increases phagocytosis and lysosomal activity in microglial cell cultures, and this was accompanied by an inhibition of inflammatory changes, and specifically inflammasome activation, induced by LPS + Aβ. Anti-TLR2 antibody triggers oxidative phosphorylation in microglia and consequently increases phagocytosis of Aβ.

## Additional files

Additional file 1: **Figure S2.** Expression of TLR2 in microglia. Microglia were prepared and treated as described in the legend for Fig. 1 and stained for TLR2 incubating with the primary antibody anti-TLR2 (Abcam, UK) followed by the secondary antibody Alexa Fluor® 488 donkey anti-mouse IgG (1:1000) and mounted in ProLong®Gold with the nuclear marker DAPI (Thermo Scientific, USA). The panel shows fluorescence images at × 40 magnification and shows that TLR2 staining is confined to the membrane of the microglial cells independently of the experimental groups observed. (Scale bar = 50 μm). (PNG 694 kb)

Additional file 2: **Figure S1.** Anti-TLR2 antibody attenuates the LPS + Aβ-induced glycolytic capacity of microglia but has no effect of LPS-induced or Aβ-induced changes. Microglia were assessed for their metabolic profile using SeaHorse technology following incubation with LPS, Aβ or both in the presence or absence of anti-TLR2 antibody as described in the “Methods” section. LPS + Aβ significantly increased mean glycolytic capacity and glycolysis (*p < 0.05). The LPS + Aβ-induced effect on glycolytic capacity was significantly attenuated when cells were also incubated with anti-TLR2 antibody (^+^*p* < 0.05; LPS + Aβ vs LPS + Aβ+anti-TLR2 antibody). The modulatory effect of the anti-TLR2 antibody on LPS + Aβ-induced glycolysis did not reach statistical significance. (PNG 67 kb)

## Abbreviations

2-DG: 2-deoxy-D-glucose
AD: Alzheimer’s disease
ANOVA: Analysis of variance
APP/PS1: Human amyloid precursor protein (APP) and presenilin 1 (PS1)
Arg1: Arginase1
ATP: Adenosine triphosphate
Aβ: Amyloid-beta
CD14, CD36, CD47, CD68: cluster of differentiation 14, 36, 47, 68
cDMEM: Dulbecco’s modified Eagle’s medium
DTT: 1,4-Dithiothreitol
ECAR: Extracellular acidification rate
FBS: Foetal bovine serum
GM-CSF: Granulocyte macrophage colony stimulating factor
IFNγ: Interferon-γ
IL-1β, IL-4, IL-6: Interleukin-1β, 4, 6
LAMP1: Lysosomal-associated membrane protein 1
LPS: Lipopolysaccharide
LTP: Long-term potentiation
M-CSF: Macrophage colony stimulating factor
mRNA: Messenger RNA
NFκβ: Nuclear factor kappa-light-chain-enhancer of activated B cells
NHS: Normal horse serum
OCR: Oxygen consumption rate
PFA: Paraformaldehyde
PFKFB1: 6-phosphofructo-2-kinase/fructose-2,6-biphosphatase 1
PFKFB3: 6-phosphofructo-2-kinase/fructose-2,6-biphosphatase
PKM2: Pyruvate kinase M2
RAGE: Receptor for advanced glycation end products
ROS: Reactive oxygen species
RT-PCR: Reverse transcription polymerase chain reaction
SEM: Standard error of the mean
TLR2: Toll-like receptor (TLR)2
TLR4: Toll-like receptor (TLR)4
TNFα: Tumour necrosis factor alpha
TREM2: Triggering receptor expressed on myeloid cells 2
VDAC: Voltage-dependent anion channel
