# Supplementary figures and images for: Anti-TLR2 antibody triggers oxidative phosphorylation in microglia and increases phagocytosis of β-amyloid
Source: J Neuroinflammation. 2018 Aug 31;15:247. doi: 10.1186/s12974-018-1281-7 (PMC6119264; doi:10.1186/s12974-018-1281-7)

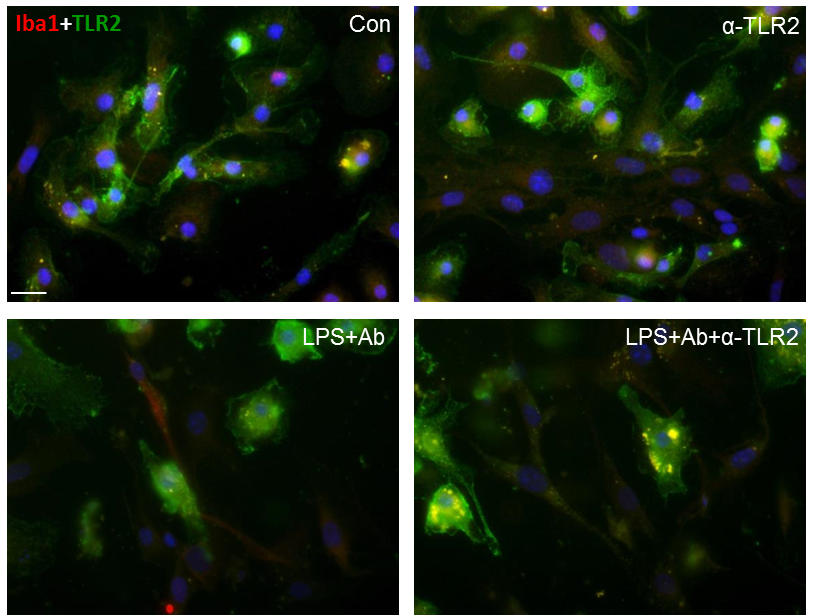

Supplement: Supplementary file 1 — Figure S2. Expression of TLR2 in microglia. Microglia were prepared and treated as described in the legend for Fig. 1 and stained for TLR2 incubating with the primary antibody anti-TLR2 (Abcam, UK) followed by the secondary antibody Alexa Fluor® 488 donkey anti-mouse IgG (1:1000) and mounted in ProLong®Gold with the nuclear marker DAPI (Thermo Scientific, USA). The panel shows fluorescence images at × 40 magnification and shows that TLR2 staining is confined to the membrane of the microglial cells independently of the experimental groups observed. (Scale bar = 50 μm). (PNG 694 kb) [file 12974_2018_1281_MOESM1_ESM.png]

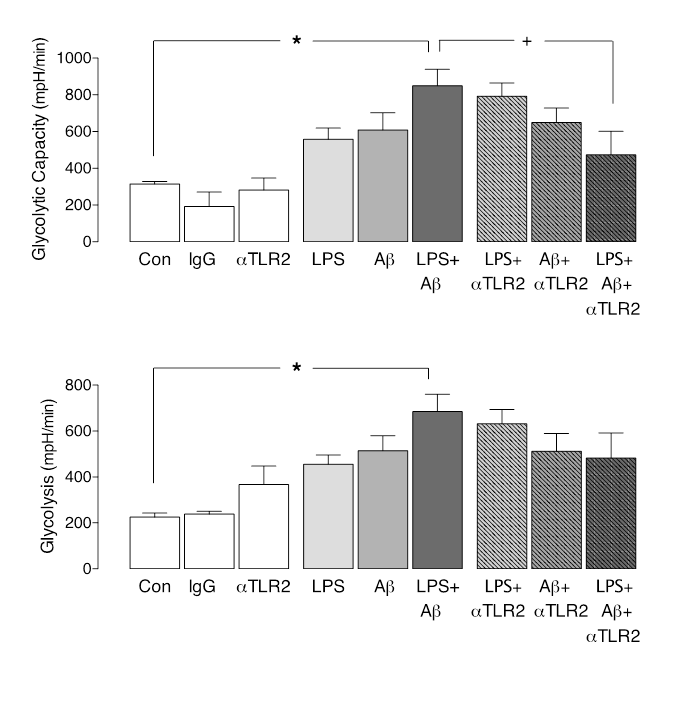

Supplement: Supplementary file 2 — Figure S1. Anti-TLR2 antibody attenuates the LPS + Aβ-induced glycolytic capacity of microglia but has no effect of LPS-induced or Aβ-induced changes. Microglia were assessed for their metabolic profile using SeaHorse technology following incubation with LPS, Aβ or both in the presence or absence of anti-TLR2 antibody as described in the “Methods” section. LPS + Aβ significantly increased mean glycolytic capacity and glycolysis (*p < 0.05). The LPS + Aβ-induced effect on glycolytic capacity was significantly attenuated when cells were also incubated with anti-TLR2 antibody (+p < 0.05; LPS + Aβ vs LPS + Aβ+anti-TLR2 antibody). The modulatory effect of the anti-TLR2 antibody on LPS + Aβ-induced glycolysis did not reach statistical significance. (PNG 67 kb) [file 12974_2018_1281_MOESM2_ESM.png]
